# Diagnostic Performance of the Fibrosis-4 Index and Nonalcoholic Fatty Liver Disease Fibrosis Score in Lean Adults With Nonalcoholic Fatty Liver Disease

**DOI:** 10.1001/jamanetworkopen.2023.29568

**Published:** 2023-08-17

**Authors:** Huiyul Park, Eileen L. Yoon, Takanori Ito, Ae Jung Jo, Mimi Kim, Jonghyun Lee, Hye-Lin Kim, Taeang Arai, Masanori Atsukawa, Miwa Kawanaka, Hidenori Toyoda, Masatoshi Ishigami, Ming-Lung Yu, Dae Won Jun, Mindie H. Nguyen

**Affiliations:** 1 Department of Family Medicine, Myoungji Hospital, Hanyang University College of Medicine, Seoul, Republic of Korea; 2 Department of Internal Medicine, Hanyang University College of Medicine, Seoul, Republic of Korea; 3 Hanyang Institute of Bioscience and Biotechnology, Hanyang University, Seoul, Republic of Korea; 4 Department of Gastroenterology and Hepatology, Nagoya University Graduate School of Medicine, Nagoya, Japan; 5 Department of Information Statistics, Andong National University, Gyeongsangbuk-do, Republic of Korea; 6 Department of Radiology, Hanyang University College of Medicine, Seoul, Republic of Korea; 7 Department of Translational Medicine, Hanyang University Graduate School of Biomedical Science and Engineering, Seoul, Republic of Korea; 8 College of Pharmacy, Sahmyook University, Seoul, Republic of Korea; 9 Division of Gastroenterology and Hepatology, Nippon Medical School, Tokyo, Japan; 10 Department of General Internal Medicine, Kawasaki Medical School General Medical Center, Okayama, Japan; 11 Department of Gastroenterology, Ogaki Municipal Hospital, Ogaki, Japan; 12 Department of Internal Medicine, Kaohsiung Medical University Hospital, Kaohsiung Medical University, Kaohsiung, Taiwan; 13 Division of Gastroenterology and Hepatology, Department of Medicine, Stanford University Medical Center, Palo Alto, California; 14 Department of Epidemiology and Population Health, Stanford University Medical Center, Palo Alto, California

## Abstract

**IMPORTANCE:**

The diagnostic performance of the fibrosis-4 index (FIB-4) and nonalcoholic fatty liver disease (NAFLD) fibrosis score (NFS) for advanced fibrosis in lean patients with NAFLD is limited.

**OBJECTIVE:**

To evaluate the diagnostic performance of the FIB-4 and NFS in lean individuals with NAFLD.

**DESIGN, SETTING, AND PARTICIPANTS:**

This diagnostic study included adults with biopsy-proven NAFLD from 6 referral centers in Asia from 1995 to 2019. Cohorts were matched by age and sex between the lean and nonlean groups. All statistical analyses were executed from October 2022 to March 2023.

**MAIN OUTCOMES AND MEASURES:**

The diagnostic performance of the FIB-4 and NFS at the current cutoff for advanced hepatic fibrosis in lean (body mass index [BMI] below 23 [calculated as weight in kilograms divided by height in meters squared]) and nonlean (BMI above 23) patients were evaluated.

**RESULTS:**

A total of 1501 patients were included in analysis (mean [SD] age, 46.1 [16.4] years); 788 male (52.5%), 115 lean (7.7%), 472 (30.2%) Korean, 821 (48.7%) Japanese, and 341 (21.3%) Taiwanese. Among the age- and sex-matched cohort, the mean (SD) age was 52.3 (15.1) years and 41.2% (47 of 114) were male. The diagnostic performance and areas under the operating characteristic curve of the FIB-4 (lean, 0.807 vs nonlean, 0.743; *P* = .28) and NFS (lean, 0.790 vs nonlean, 0.755; *P* = .54) between the 2 groups were comparable in the age- and sex-matched cohort. The sensitivity and specificity of the NFS showed increasing and decreasing tendency according to the BMI quartiles (*P* for trend < .001), while those of the FIB-4 did not (*P* for trend = .05 and *P* = .20, respectively). Additionally, although the areas under the operating characteristic curve of the FIB-4 and NFS were not significantly different in the lean group (0.807 vs 0.790; *P* = .09), the sensitivity of the current NFS cutoff values was lower in the lean group than in that of FIB-4 (54.4% vs 81.8%; *P* = .03).

**CONCLUSIONS AND RELEVANCE:**

In this cohort study, the performance of the FIB-4 and NFS in diagnosing advanced fibrosis did not differ significantly between the 2 groups overall. However, in lean NAFLD, while the sensitivity for diagnosing advanced hepatic fibrosis remained reasonable at the current cutoff level, the sensitivity of NFS at the current cutoff was too low to be an adequate screening tool.

## Key Points

**Question:**

Is it appropriate to apply conventional fibrosis-4 index (FIB-4) and nonalcoholic fatty liver disease (NAFLD) fibrosis score (NFS) cutoffs for assessing advanced fibrosis in lean adults with NAFLD?

**Findings:**

In this diagnostic study including age- and sex-matched adults with NAFLD, the overall diagnostic performance of FIB-4 and NFS were not significantly different between the lean and nonlean groups. However, while the sensitivity and specificity of FIB-4 were not affected by body mass index and remained reasonable, those of NFS were unacceptably low to be used as first step screening tool.

**Meaning:**

These findings suggest that the FIB-4, at the current cutoff value, represents a better screening parameter of advanced NAFLD fibrosis in lean individuals.

## Teaser

This diagnostic study of adults in Asia evaluates the performance of 2 index scores in estimating clinical outcomes for individuals with nonalcoholic fatty liver disease.

## Introduction

Nonalcoholic fatty liver disease (NAFLD) is the leading cause of chronic liver disease and the second most common etiology of hepatocellular carcinoma and liver transplantation in most Western countries.^1,2^ In NAFLD, the stage of hepatic fibrosis is a critical risk factor for estimating clinical hard outcomes (eg, overall and liver-related mortality). Therefore, algorithms for screening high-risk groups for advanced hepatic fibrosis in patients with NAFLD are crucial.

The diagnostic performance of the fibrosis-4 index (FIB-4) and NAFLD fibrosis score (NFS) have been extensively studied in patients with NAFLD.^3,4^ Although the specificity and positive predictive value of FIB-4 and NFS for advanced hepatic fibrosis are low, these 2 measures demonstrate high negative predictive values and acceptable sensitivity. Currently, most practice guidelines recommend using the FIB-4 and NFS as the initial step for selecting high-risk groups among patients with obesity who have NAFLD.^5,6^

Lean patients (body mass index [BMI] below 25 in Western and 23 in Eastern countries; calculated as weight in kilograms divided by height in meters squared) account for between approximately 15% to 19% of patients with NAFLD.^7,8^ The American Gastroenterological Association (AGA) Institute’s Clinical Practice Updates Committee and Governing Board recommended the use of serum noninvasive tests (FIB-4 and NFS) as the first step in stratifying liver fibrosis risk in lean patients with NAFLD.^6^ However, the diagnostic performance and adequate cutoffs for the FIB-4 and NFS in lean individuals with NAFLD have not been evaluated. In this diagnostic study, we attempted to evaluate the diagnostic performance and appraise the current cutoff values of the FIB-4 and NFS in lean individuals with NAFLD.

## Methods

Patients with biopsy-proven NAFLD were included from 6 referral centers in Japan, Taiwan, and Korea from 1995 to 2019 (information on hepatic fibrosis assessment and diagnosis of NAFLD is available in eMethods in Supplement 1). The study protocol is in accordance with the principles of both the Declarations of Helsinki and Istanbul Helsinki,^19^ and was approved by the institutional review board of Hanyang University Hospital. The ethics committee waived the requirement for written informed consent for participation owing to the retrospective nature of the study. For analysis, we used anonymous clinical data to reduce the risk of bias. This study followed the Standards for Reporting of Diagnostic Accuracy (STARD) reporting guideline.

### Study Population

**Figure 1.**
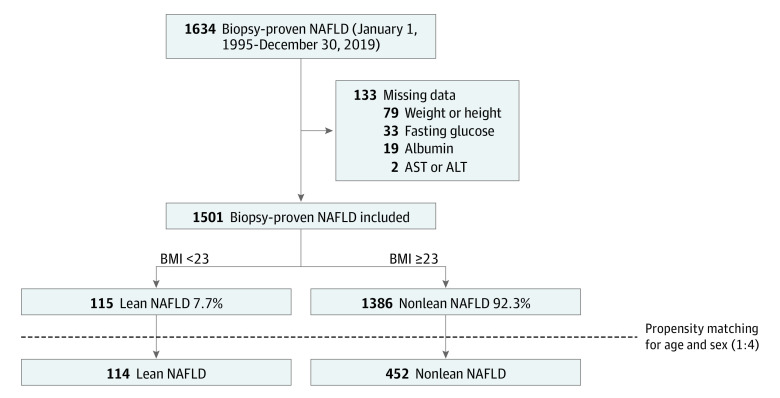
Study Flow AST indicates aspartate transaminase; ALT, alanine transaminase; BMI, body mass index (calculated as weight in kilograms divided by height in meters squared); NAFLD, nonalcoholic fatty liver disease.

Patients with NAFLD who underwent liver biopsy were included for assessment, among whom those missing clinical information (eg, FIB-4 and NFS variables) were excluded (Figure 1). Individuals with a BMI cutoff of 23 were categorized as lean based on the World Health Organization guidelines for the Asia-Pacific region^9^ and the Korean Society for the Study of Obesity criteria.^10^

### Propensity Score Matching (Age and Sex)

Propensity score matching was performed to reduce the imbalances of sample size and confounding factors between the lean and nonlean NAFLD groups. Age and sex were used as covariates to calculate propensity scores. After exact-matching for sex, the propensity score was evaluated by hierarchically fitting the sex and age to the logistic regression model. The allowed limit was 0.1-times the logit of the propensity score calculated using a caliper. Greedy matching method was applied using nearest neighbor 1:4 matching. SAS version 9.4 (SAS Institute Inc) was used for the analysis. The baseline characteristics and age distribution before and after propensity score matching in lean and nonlean patients with NAFLD are summarized in the Table and eFigure in Supplement 1.

**Table.** Baseline Characteristics of Patients With Biopsy-Proven NAFLD With and Without Age- and Sex-Matching

Characteristics	Total, No. (%) (N = 1501)	Without age and sex matching, No. (%)		*P* value	With age and sex matching, No. (%)		*P* value
		Lean NAFLD (n = 115)	Nonlean NAFLD (n = 1386)		Lean NAFLD (n = 114)	Nonlean NAFLD (n = 452)	
Age, mean (SD), y	46.1 (16.4)	52.5 (15.6)	45.6 (16.3)	<.001	52.2 (15.4)	52.2 (15.1)	.96
Sex							
Male	788 (52.5)	48 (41.7)	740 (53.4)	.01	47 (41.2)	186 (41.2)	.98
Female	713 (47.5)	67 (58.3)	646 (46.6)		67 (58.8)	266 (58.8)	
BMI, mean (SD)	29.9 (6.7)	21.3 (1.6)	30.6 (6.4)	<.001	21.2 (1.6)	29.0 (4.7)	<.001
Glucose, mean (SD), mg/dL	113 (36)	115 (43)	113 (35)	.51	116 (42)	117 (32)	.82
Impaired fasting glucose	870 (58.0)	61 (53.0)	809 (58.4)	.26	61 (53.5)	306 (67.7)	.005
Diabetes	538 (35.8)	33 (28.7)	505 (36.4)	.09	32 (28.1)	200 (44.2)	.002
AST, mean (SD), IU/L	64 (55)	59 (50)	65 (55)	.24	59 (50)	67 (48)	.12
ALT, mean (SD), IU/L	88 (77)	70 (70)	89 (78)	.01	71 (70)	85 (70)	.05
AST/ALT ratio, mean (SD)	0.90 (0.54)	1.00 (0.47)	0.89 (0.55)	.03	1.00 (0.47)	0.94 (0.51)	.23
Albumin, mean (SD), g/dL	4.31 (0.44)	4.26 (0.61)	4.31 (0.42)	.24	4.2 (0.6)	4.2 (0.4)	.59
Platelets, mean (SD), ×10^9^/L	236 (83)	213 (75)	238 (84)	.002	213 (76)	221 (69)	.27
FIB-4 index, mean (SD)	1.66 (1.47)	2.11 (1.73)	1.62 (1.44)	.001	2.11 (1.74)	2.07 (1.67)	.84
NAFLD fibrosis score, mean (SD)	–1.52 (1.74)	–1.72 (1.75)	–1.51 (1.73)	.20	–1.73 (1.75)	–1.03 (1.61)	<.001
Pathologic diagnosis							
Unknown or borderline	126 (8.4)	12 (10.4)	114 (8.2)	NA	12 (10.5)	38 (8.4)	NA
NAFLD	330 (22.0)	40 (34.8)	290 (20.9)		39 (34.2)	69 (15.3)	
NASH	1045 (69.6)	63 (54.8)	982 (70.9)		63 (55.3)	345 (76.3)	
Liver fibrosis by histology							
F0	252 (16.8)	30 (26.1)	222 (16.0)	.03	30 (26.3)	61 (13.5)	.002
F1	586 (39.0)	45 (39.1)	541 (39.0)		45 (39.5)	155 (34.3)	
F2	351 (23.4)	17 (14.8)	334 (24.1)		17 (14.9)	115 (25.4)	
F3	239 (15.9)	18 (15.7)	221 (15.9)		18 (15.8)	92 (20.4)	
F4	73 (4.9)	5 (4.3)	68 (4.9)		4 (3.5)	29 (6.4)	
Significant fibrosis (≥F2)^a^	663 (44.2)	40 (34.8)	623 (44.9)	.03	39 (34.2)	236 (52.2)	.001
Advanced fibrosis (≥F3)^a^	312 (20.8)	23 (20.0)	289 (20.9)	.82	22 (19.3)	121 (26.8)	.10

Abbreviations: AST, aspartate transaminase; ALT, alanine transaminase; BMI, body mass index (calculated as weight in kilograms divided by height in meters squared); FIB-4 index, fibrosis-4 index; NAFLD, nonalcoholic fatty liver disease; NASH, nonalcoholic steatohepatitis.

To convert glucose to millimoles per liter, multiply by 0.0555; AST to microkatal per liter, multiply by 0.0167; ALT to microkatal per liter, multiply by 0.0167; albumin to gram per liter, multiply by 10; platelets to ×10^9^ per liter, multiply by 1.0.

^a^ Significant fibrosis or advanced fibrosis was defined as fibrosis stage 2 through 4 (F2-F4) or stage 3 and 4 (F3-F4), respectively (eMethods in Supplement 1).

### FIB-4 Index and NFS

The FIB-4 and NFS were calculated, and the low cutoff values of FIB-4 and NFS were selected as described by McPherson et al.^11^ If the patients were aged 65 years or older, the cutoff values of 2.0 and 0.12 were used for FIB-4 and NFS, whereas if they were aged under 65 years, the cutoff values of 1.3 and –1.455 were used.

### Statistical Analysis

Continuous and categorical variables are presented as mean averages and numbers and percentages, respectively. Categorical variables were analyzed using either the χ^2^ or Fisher exact test, whereas continuous variables were analyzed using the Student independent *t* test. Statistical analyses were performed using SPSS for Windows version 26.0 (SPSS Inc). The area under the receiver operating characteristic curves (AUROC) of FIB-4 and NFS were compared using the DeLong test in MedCalc version 20 (MedCalc Software Ltd). The statistical difference of sensitivity, specificity, positive predictive value (PPV), and negative predictive value (NPV) of the FIB-4 and NFS between the lean and nonlean groups or according to BMI quartiles were analyzed using χ^2^ test. Mann-Whitney *U* test was used in cases where the sample number of the group was insufficient. These analyses were performed using R Statistical Software version 2.14.0 (R Foundation for Statistical Computing). Statistical significance was set at *P* < .05 in 2-sided tests.

## Results

### Baseline Characteristics

A total of 1501 patients with biopsy-proven NAFLD were included in the study. The mean (SD) age of the included patients was 46.1 (16.4) years, and 788 (52.5%) were male. This cohort consisted of 472 Korean (30.2%), 821 Japanese (48.7%), and 341 Taiwanese (21.1%) people. The proportion of lean patients in the biopsy-proven NAFLD cohort was 7.7% (115 patients) (Figure 1). The mean (SD) BMI of lean individuals with NAFLD was significantly lower than that of nonlean patients (21.3 [1.6] vs 30.6 [6.4]; *P* < .001) (Table). Lean patients with NAFLD were older and more likely to be female than nonlean patients with NAFLD. The prevalence of significant and advanced hepatic fibrosis in the lean group was 34.8% (40 of 115) and 20.0% (23 of 115), respectively, with no significant difference in the prevalence of advanced hepatic fibrosis between the lean and nonlean patients with NAFLD (23 of 115 [20.0%] vs 289 of 1386 [20.9%]; *P* = .82) (Table).

Age and sex matching was performed at a 1:4 ratio to balance the background differences between the lean and nonlean NAFLD groups; this yielded a matched cohort of 114 lean and 452 nonlean patients with NAFLD (233 male [41.2%]) whose mean (SD) age was 52.3 (15.1) years. Mean (SD) BMI of lean individuals with NAFLD was significantly lower than that of nonlean patients in the age- and sex-matched cohort (21.2 [1.6] vs 29.0 [4.7]; *P* < .001). The prevalence of advanced hepatic fibrosis in lean and nonlean patients with NAFLD were 19.3% (22 of 114) and 26.8% (121 of 452), respectively.

### Diagnostic Performances of the FIB-4 and NFS for Advanced Fibrosis

**Figure 2.**
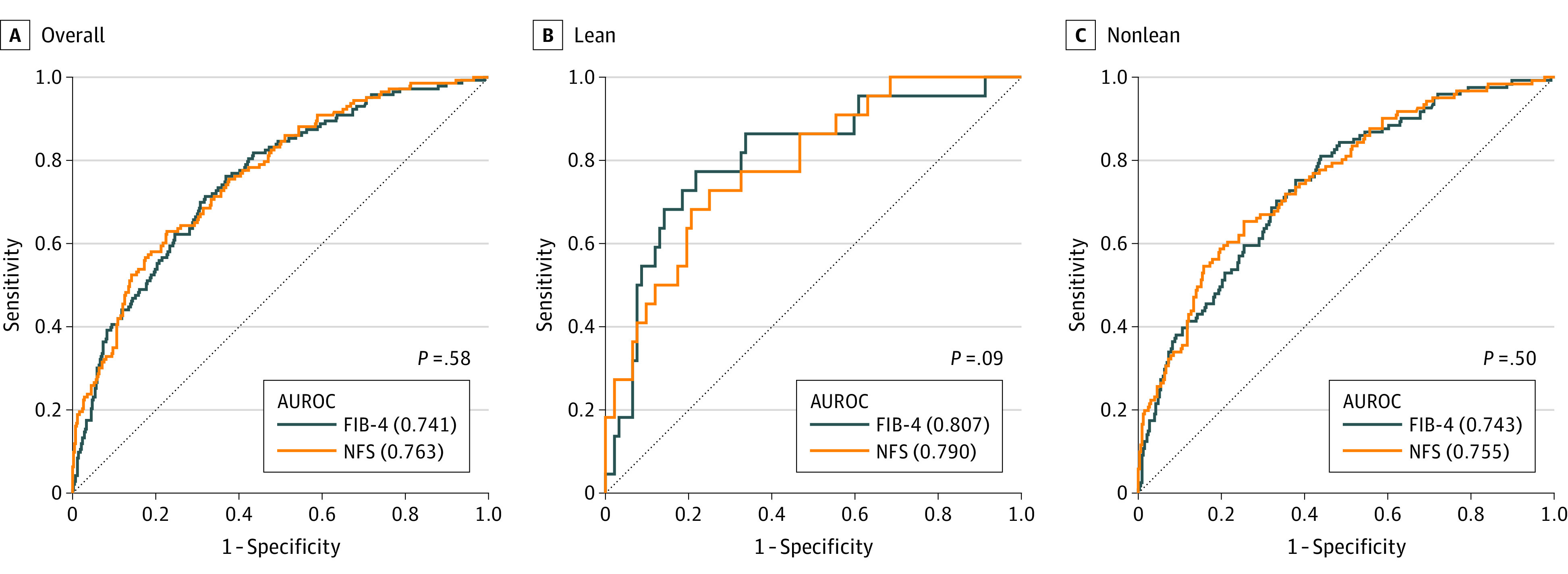
Comparison of the Area Under the Receiver Operating Characteristic Curve (AUROC) Between the Fibrosis-4 Index (FIB-4) and Nonalcoholic Fatty Liver Disease Fibrosis Score (NFS) in an Age- and Sex-Matched Cohort *P* values for the comparison of FIB-4 with NFS.

Although the AUROC of the FIB-4 for advanced fibrosis was higher than that of the NFS overall before adjustment for sex and age (FIB-4, 0.766 vs NFS, 0.738; *P* = .006), there was no significant difference in AUROC between FIB-4 and NFS after adjusting for sex and age (FIB-4, 0.754 vs NFS, 0.763; *P* = .58) (eTable 1 in Supplement 1; Figure 2A). The AUROCs of the FIB-4 and NFS were comparable in nonlean patients with NAFLD (FIB-4, 0.743 vs NFS, 0.755; *P* = .50) (Figure 2C) and in lean patients with NAFLD (FIB-4, 0.807 vs NFS, 0.790; *P* = .09) (Figure 2B).

Notably, the diagnostic performance and AUROCs of the FIB-4 and NFS were comparable between the lean and nonlean groups. The AUROCs of the FIB-4 were comparable between the lean and nonlean groups (lean, 0.807 vs nonlean, 0.743; *P* = .28) (Figure 2B and C), while the AUROCs of the NFS were also comparable between the lean and nonlean NAFLD cohort (lean, 0.790 vs nonlean, 0.755; *P* = .54). The accuracy of the FIB-4 and NFS in diagnosing advanced hepatic fibrosis was comparable, regardless of the BMI group (eTable 1 in Supplement 1).

### Sensitivity and Specificity of FIB-4 in Lean NAFLD

**Figure 3.**
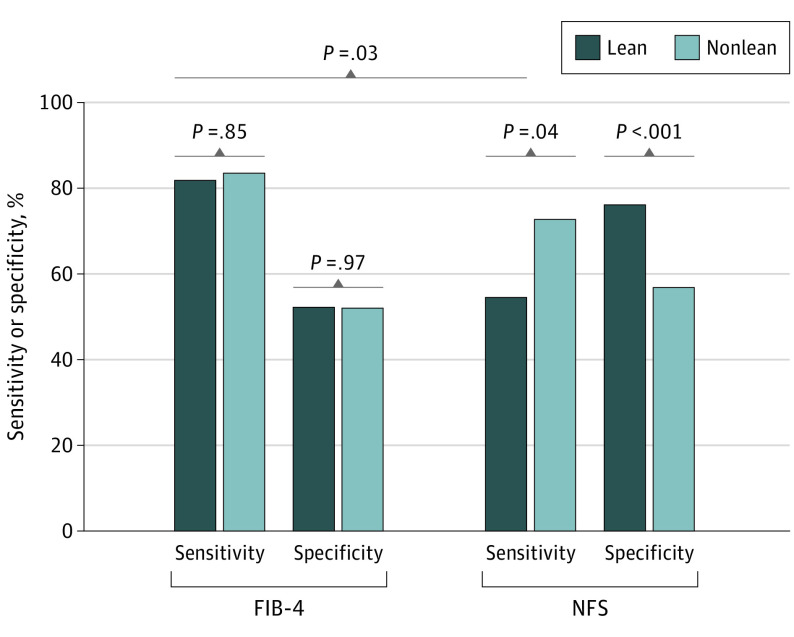
Comparison of the Sensitivity and Specificity of the Fibrosis-4 Index (FIB-4) and Nonalcoholic Fatty Liver Disease Fibrosis Score (NFS) in an Age- and Sex-Matched Cohort Comparison of the performances (sensitivity and specificity) of the FIB-4 and NFS for diagnosing patients with advanced hepatic fibrosis by using their lower cutoff values between the lean and nonlean patients in an age- and sex-matched cohort.

The sensitivity, specificity, and positive and negative predictive values using the current age-adjusted cutoff values of FIB-4 and NFS for diagnosis are summarized in eTable 1 in Supplement 1 and in Figure 3. In the age- and sex-matched cohort, no significant differences were observed in the sensitivity (81.8% vs 83.5%; *P* = .85) and specificity (52.2% vs 52.0%; *P* = .97) of FIB-4 between the lean and nonlean groups (Figure 3). The NFS had a lower sensitivity (54.5% vs 72.7%; *P* = .04) and higher specificity (76.1% vs 56.8%; *P* < .001) in lean patients with NAFLD than in nonlean patients. Moreover, the sensitivity of the NFS in lean patients with NAFLD was lower than that of the FIB-4 (54.5% vs 81.8%; *P* = .03). Interestingly, a similar pattern was also observed between the diabetic and nondiabetic groups. The NFS showed lower sensitivity in patients with NAFLD without diabetes compared with those with diabetes (59.7% vs 78.9%; *P* = .02), while there was no statistically significant difference in the sensitivity of FIB-4 between patients with and without diabetes (eTable 2 in Supplement 1).

**Figure 4.**
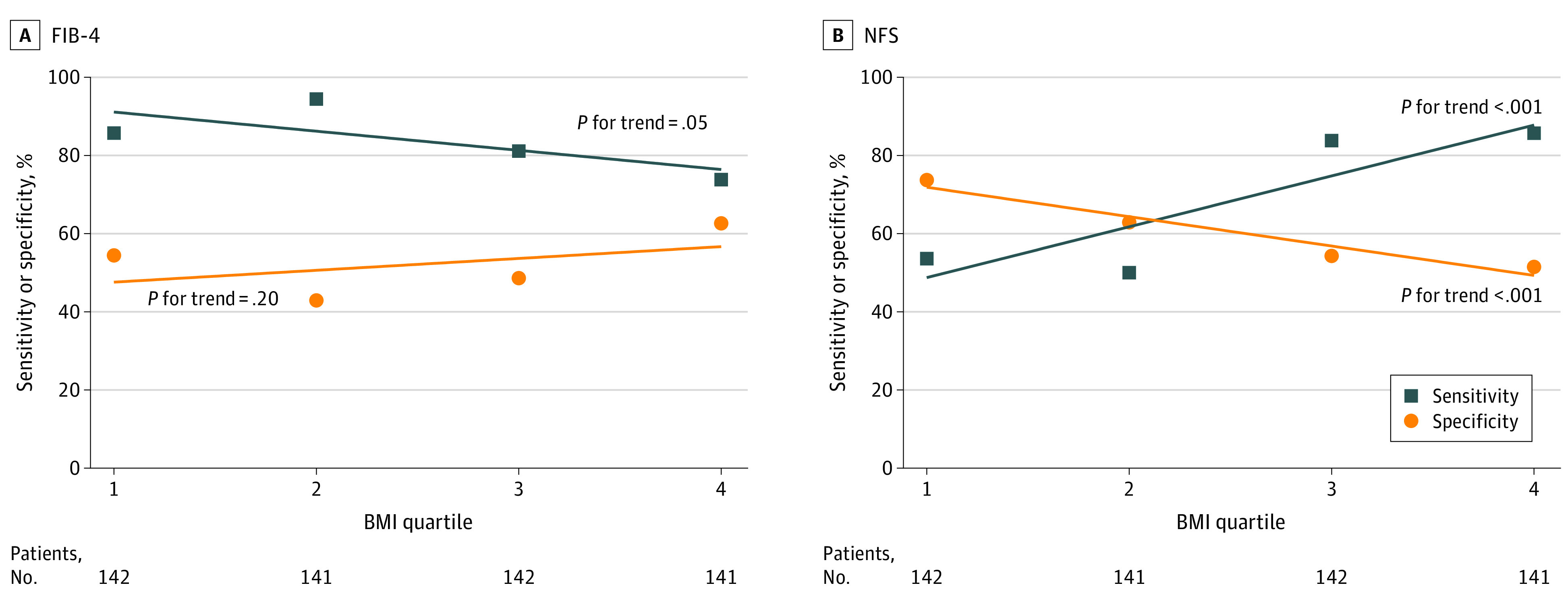
Sensitivity and Specificity Trend of Fibrosis-4 Index (FIB-4) and Nonalcoholic Fatty Liver Disease Fibrosis Score (NFS) According to Body Mass Index (BMI) Quartiles

The sensitivity and specificity of the FIB-4 and NFS using the current age-specific cutoff values according to BMI quartiles were plotted in age- and sex-matched cohorts (Figure 4A and B). The difference in sensitivity (85.7% to 73.8%; *P* for trend = .05) and specificity (54.4% to 62.6%; *P* for trend = .20) of the FIB-4 according to increasing BMI was not significant. However, the sensitivity of the NFS increased (53.6% to 85.7%; *P* for trend < .001) with increasing BMI, while the specificity decreased (73.7% to 51.5%; *P* for trend < .001) with increasing BMI.

## Discussion

Our study evaluated the FIB-4 and NFS in lean patients with NAFLD for diagnosing liver fibrosis. The performance of the FIB-4 and NFS between the lean and nonlean groups was compared in large-scale, multicenter, Asian cohorts with biopsy-confirmed NAFLD. Although the AUROC and diagnostic accuracy of the FIB-4 and NFS were comparable, the sensitivity and specificity of the NFS for diagnosing advanced hepatic fibrosis at the current cutoff tended to increase and decrease, respectively, as the BMI of the target patients increased. Therefore, one should take care when applying the NFS to lean patients with NAFLD using the current cutoff because of the lower sensitivity. Several studies have evaluated the diagnostic performance of the FIB-4 and NFS according to various patient characteristics, such as age, BMI, and comorbidities, among which age was identified as a strong confounding factor in evaluation of the hepatic stiffness degree.^11,12,13,14,15,16^ Therefore, age-specific cutoff values are currently used for the 2 noninvasive tests (NITs). Our data also showed that BMI itself did not affect the diagnostic performance of the FIB-4 index and NFS. However, careful attention should be paid to the cutoff values when using NFS in lean patients.

Relatively few BMI-related studies have been conducted compared with those focused on other factors. A validation study of the performance of the FIB-4 and NFS in diagnosing advanced fibrosis in all BMI categories was conducted in 2022.^12^ However, that study failed to evaluate the AUROC of the FIB-4 and NFS for diagnosing advanced hepatic fibrosis in lean patients with NAFLD. Additionally, the study highlighted the low accuracy of the FIB-4 and NFS for diagnosing advanced fibrosis, especially in lean patients with NAFLD.^12,17^ This may have been due to the small sample size (28 patients) in their study compared with the AUROCs of the FIB-4 and NFS of the cohorts our study, as well as in another with a larger sample size.^13,16^

Another previous study evaluated the diagnostic performance of various hepatic fibrosis scores in a cohort^13^ consisting of 709 patients without obesity with biopsy-proven NAFLD from 3 centers. The authors reported that both the FIB-4 and NFS for liver fibrosis performed well in patients without obesity (with BMI below 25). In line with this study, 2022 AGA practice updates have recommended the use of either the FIB-4 or NFS as a first-step tool for the risk assessment of hepatic fibrosis in lean patients with NAFLD (BMI below 25 and 23 in Western and Asian countries, respectively).^6^ However, their diagnostic performance in lean patients with NAFLD (BMI below 23) was not compared because only 81 patients did not have obesity. To our knowledge, our study stands out as the first to compare the diagnostic performance of the FIB-4 and NFS in a large cohort of lean individuals with liver biopsy-confirmed NAFLD. Our findings are similar to those of previous reports. Although the diagnostic performance (AUROCs) of the NFS was similar between patients without and with obesity (0.85 vs 0.81), the sensitivity was lower in patients without than in those with obesity (0.67 vs 0.81). The AUROC (0.86 vs 0.73), sensitivity (0.77 vs 0.73), and specificity (0.77 vs 0.64) of the FIB-4 was similar between the patients without and with obesity with NAFLD^13^; our data showed similar results. The FIB-4 and NFS showed similar diagnostic performances (AUROC) depending on the BMI. However, in lean patients with NAFLD, the FIB-4 was a more reliable parameter than the NFS when the current cutoff value was applied. This finding indicates that both the FIB-4 and NFS have good diagnostic performances for advanced hepatic fibrosis; however, the currently recommended cutoff value for the NFS may need to be changed for lean patients.

In this study, the mean NFS value in nonlean patients was significantly higher than that in lean patients in the age- and sex-matched cohort (Table). However, there was no statistically significant difference in the prevalence of advanced hepatic fibrosis between the 2 groups. Additionally, the mean NFS value increased as the BMI quartiles increased (eTable 3 in Supplement 1). In contrast, the mean FIB-4 value in nonlean patients did not differ significantly from that in lean patients.

These findings suggest that the NFS may either underestimate or overestimate the degree of hepatic fibrosis depending on the BMI distribution of the target population. One key difference between the NFS and FIB-4 is the inclusion of abnormal glucose levels and BMI as components of formulae in NFS. Therefore, the NFS is inevitably affected by the presence or absence of these factors. Consequently, the sensitivity and specificity of the NFS varied at the currently accepted conventional cutoff values with changes in BMI (Figure 4B). Moreover, the NFS showed lower sensitivity in patients with NAFLD without diabetes compared with those with diabetes (59.7% vs 78.9%; *P* = .02), while there was no statistically significant difference in the sensitivity of FIB-4 between patients with and without diabetes (eTable 2 in Supplement 1).

Considering that there was no significant difference in the AUROCs between the FIB-4 and NFS for diagnosing advanced hepatic fibrosis across all BMI phenotypes (Figure 2), the diagnostic capacity of the NFS may not be inferior to that of FIB-4. However, the sensitivity (54.5%) of the NFS in the lean NAFLD group implies that it is possible to miss the 45.5% of lean patients with NAFLD with advanced hepatic fibrosis at the first screening step. Therefore, it will be necessary to lower the cutoff value of the NFS to increase its sensitivity to be comparable with that in nonlean patients with NAFLD or that of the FIB-4 (approximately 70% to 80%). However, the use of different cutoff values according to the BMI condition represents a significant hurdle to the clinical utilization of NFS. Therefore, we believe that the FIB-4 is more appropriate than the NFS and thus, should be used as a first-step screening tool for advanced hepatic fibrosis, regardless of the BMI condition.

The diagnostic performance (AUROC) of the FIB-4 and NFS in our study was similar to that of previous studies. Although the AUROC of the FIB-4 was higher than that of the NFS in the overall patients before adjustment for sex and age (FIB-4, 0.766 vs NFS, 0.738; *P* = .006), there was no significant difference in AUROC between FIB-4 and NFS after adjusting for sex and age (FIB-4, 0.754 vs NFS, 0.763; *P* = .58) (eTable 1 in Supplement 1; Figure 2A); this indicates that the patient’s age distribution has an effect on the diagnostic performance of these 2 NITs. Moreover, an adequate number of lean patients with NAFLD is crucial for a precise analysis considering that the AUROC evaluation for diagnosing advanced fibrosis failed in a previous study.^12^ Adjustment for age and adequate sample size are significant factors for the diagnostic performance evaluation of these NITs in lean patients with NAFLD. In this regard, we believe that our study yields the most reliable results among recent relevant studies.

### Limitations

This study has several limitations. First, we did not consider the effect of age when evaluating the sensitivity and specificity of the FIB-4 and NFS according to BMI quartiles (Figure 4). Moreover, fasting glucose level and diabetes, which are the relevant variables in calculating the FIB-4 and NFS, were not considered in propensity matching between the lean and nonlean groups. However, the fasting glucose level and diabetes were not significantly different according to BMI quartiles (eTable 3 in Supplement 1). The mean age of patients in the highest BMI group was the lowest among the 4 BMI groups. Moreover, our cohort had a similar rate of advanced hepatic fibrosis according to BMI quartiles, which is a strength in the assessment of diagnostic performance and interpretation. Therefore, we believe that our results are reliable. Nevertheless, future studies that assess the diagnostic performance of the FIB-4 and NFS according to BMI quartiles in the age-adjusted state are required. Second, as the cohort analyzed in this study consists of Asian patients only, the results do not represent the global population. Therefore, a validation study in patients of other races and ethnicities is needed. Third, there can be discordance in the liver histology interpretation among pathologists. However, among patients with NAFLD, prior studies have indicated histologic interpretation discordance mainly for the assessment of hepatic inflammation and ballooning but not for fibrosis, which is the primary outcome of our study.^18^

## Conclusions

In this cohort study, the AUROCs and overall diagnostic accuracy of the FIB-4 and NFS for advanced fibrosis did not differ significantly between lean and nonlean patients with NAFLD. However, while the sensitivity and specificity of the FIB-4 for advanced fibrosis were not affected by BMI, the sensitivity of the NFS for advanced fibrosis in lean patients with NAFLD was poor to the point where 1 in 2 patients with advanced fibrosis may be missed. Thus, the NFS at the conventional cutoff values should not be used to screen for fibrosis in lean patients with NAFLD.

## Notes

Supplement 1.
eMethods.
**eFigure.** Comparison of Age Distribution **eTable 1.** Comparison of the Diagnostic Performance of the FIB-4 and NFS for Diagnosing Individuals With Advanced Hepatic Fibrosis Between Lean and Nonlean Individuals **eTable 2.** Comparison of the Diagnostic Performance of the FIB-4 and NFS for Diagnosing Individuals With Advanced Hepatic Fibrosis at Current Cut-Off Values Between Nondiabetic and Diabetic Individuals **eTable 3.** Baseline Characteristics of Patients With Biopsy-Proven NAFLD in an Age- and Sex-Matched Cohort According to BMI Quartiles
eReferences

Supplement 2.
Data Sharing Statement
