# Error in Byline

**DOI:** 10.1001/jamanetworkopen.2023.36365

**Published:** 2023-09-18

In the Original Investigation titled “Diagnostic Performance of the Fibrosis-4 Index and Nonalcoholic Fatty Liver Disease Fibrosis Score in Lean Adults With Nonalcoholic Fatty Liver Disease,”^1^ published August 17, 2023, there was an error in the spelling of a coauthor’s name. This article has been corrected.^1^
