# Supplementary material for: Diagnostic Performance of the Fibrosis-4 Index and Nonalcoholic Fatty Liver Disease Fibrosis Score in Lean Adults With Nonalcoholic Fatty Liver Disease
Source: JAMA Netw Open. 2023 Aug 17;6(8):e2329568. doi: 10.1001/jamanetworkopen.2023.29568 (PMC10436134; doi:10.1001/jamanetworkopen.2023.29568)
Supplement: Supplement 1. — eMethods. eFigure. Comparison of Age Distribution eTable 1. Comparison of the Diagnostic Performance of the FIB-4 and NFS for Diagnosing Individuals With Advanced Hepatic Fibrosis Between Lean and Nonlean Individuals eTable 2. Comparison of the Diagnostic Performance of the FIB-4 and NFS for Diagnosing Individuals With Advanced Hepatic Fibrosis at Current Cut-Off Values Between Nondiabetic and Diabetic Individuals eTable 3. Baseline Characteristics of Patients With Biopsy-Proven NAFLD in an Age- and Sex-Matched Cohort According to BMI Quartiles eReferences [file jamanetwopen-e2329568-s001.pdf]

Supplemental Online Content

Park H, Yoon EL, Ito T, et al. Diagnostic performance of the Fibrosis-4 index and nonalcoholic fatty liver disease fibrosis score in lean adults with nonalcoholic fatty liver disease. *JAMA Netw Open.* 2023;6(8):e2329568. doi:10.1001/jamanetworkopen.2023.29568

eMethods.

eFigure 1. Comparison of Age Distribution

eTable 1. Comparison of the Diagnostic Performance of the FIB-4 and NFS for Diagnosing Individuals With Advanced Hepatic Fibrosis Between Lean and Nonlean Individuals

eTable 2. Comparison of the Diagnostic Performance of the FIB-4 and NFS for Diagnosing Individuals With Advanced Hepatic Fibrosis at Current Cut-Off Values Between Nondiabetic and Diabetic Individuals

eTable 3. Baseline Characteristics of Patients With Biopsy-Proven NAFLD in an Age- and Sex-Matched Cohort According to BMI Quartiles

eReferences

This supplemental material has been provided by the authors to give readers additional information about their work.

© 2023 Park H et al. *JAMA Network Open.*

eMethods

Hepatic fibrosis assessment

In this study, indications for liver biopsy to confirm a diagnosis of NAFLD were determined by each attending doctor on the basis of clinical data, including elevated liver transaminase, fibrosis tests, and the presence of metabolic factors. Patients whose liver biopsy specimens showed steatosis or steatosis with nonspecific inflammation without features of nonalcoholic steatohepatitis (NASH) were identified as nonalcoholic fatty liver (NAFL).[1] The stages of hepatic fibrosis were defined as follows: 1) stage 1, zone 3 perisinusoidal fibrosis; 2) stage 2, zone 3 perisinusoidal fibrosis with portal fibrosis; 3) stage 3, zone 3 perisinusoidal and portal fibroses with bridging fibrosis; and 4) stage 4, cirrhosis. The assessment of hepatic fibrosis stages followed the same standard in all referral centers. Significant fibrosis or advanced fibrosis was defined as stage 2–4 (F2–4) or stage 3–4 (F3–4), respectively.

Diagnosis of NAFLD

Patients with NAFLD were diagnosed according to the AASLD, EASL-EASD-EASO, and KASL guidelines.[1-3] We confirmed the following: 1) presence of liver steatosis on imaging; 2) absence of excessive alcohol use (ethanol intake < 210 g per week for men and < 140 g per week for women); 3) no other causes of fatty liver, such as drugs; and 4) no other causes of chronic liver disease, such as viral infection (hepatitis C or B virus), primary biliary cholangitis, or autoimmune hepatitis. All of the included patients underwent percutaneous liver biopsy. The indications for liver biopsy were determined by each attending doctor based on clinical data, including elevated liver transaminase levels, fibrosis tests, and the presence of metabolic factors.

© 2023 Park H et al. *JAMA Network Open.*

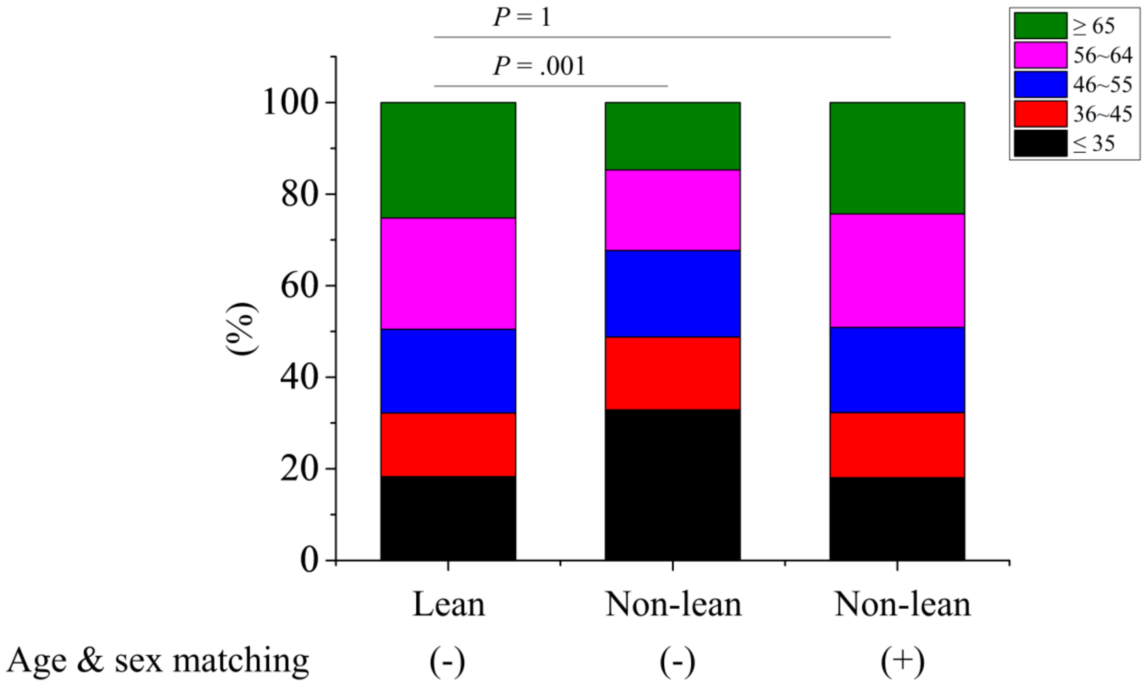

eFigure 1. Comparison of Age Distribution

Comparison of the proportion of age between lean and non-lean subjects with and without age and sex matching.

© 2023 Park H et al. *JAMA Network Open.*

eTable 1. Comparison of the Diagnostic Performance of the FIB-4 and NFS for Diagnosing Individuals With Advanced Hepatic Fibrosis Between Lean and Nonlean Individuals

	AUROCs (95% CI)	Sensitivity (95% CI)	Specificity (95% CI)	PPV (95% CI)	NPV (95% CI)	Accuracy (95% CI)
a) FIB-4/age and sex matching (–)						
Overall patients	0.766 (0.736–0.796)	76.3 (71.6–80.5)	64.9 (63.7– 66.0)	36.3 (34.1–38.2)	91.3 (89.5–92.8)	67.3 (65.4–69.0)
Lean NAFLD	0.806 (0.704–0.909)	82.6 (62.6–94.1)	52.2 (47.2–55.1)	30.2 (22.9–34.4)	92.3 (83.5–97.4)	58.3 (50.3–62.9)
Non-lean NAFLD	0.765 (0.734–0.796)	75.8 (70.9–80.2)	66.0 (64.7–67.2)	37.0 (34.6–39.1)	91.2 (89.4–92.8)	68.0 (66.0–69.9)
b) NFS/age and sex matching (–)						
Overall patients	0.738 (0.705–0.770)	63.8 (58.8–68.5)	64.7 (63.4–65.9)	32.1 (29.6–34.6)	87.2 (85.4–88.9)	64.5 (62.4–66.5)
Lean NAFLD	0.792 (0.694–0.890)	52.2 (33.0–70.5)	76.1 (71.3–80.7)	35.3 (22.3–47.7)	86.4 (81.0–91.6)	71.3 (63.6–78.6)
Non-lean NAFLD	0.734 (0.700–0.768)	64.7 (59.5–69.6)	63.7 (62.4–65.0)	32.0 (29.4–34.4)	87.3 (85.4–89.0)	63.9 (61.8–66.0)
c) FIB-4/age and sex matching (+)						
Overall patients	0.754 (0.709–0.800)	83.2 (76.6–88.5)	52.0 (49.8–53.8)	37.0 (34.0–39.3)	90.2 (86.3–93.2)	59.9 (56.6–62.5)
Lean NAFLD	0.807 (0.701–0.913)	81.8 (61.2–93.9)	52.2 (47.2–55.1)	29.0 (21.7–33.3)	92.3 (83.6–97.4)	57.9 (49.9–62.5)
Non-lean NAFLD	0.743 (0.693–0.793)	83.5 (76.3–89.1)	52.0 (49.4–54.0)	38.8 (35.5–41.5)	89.6 (85.1–93.1)	60.4 (56.6–63.4)
d) NFS/age and sex matching (+)						
Overall patients	0.763 (0.719–0.808)	69.9 (62.7–76.5)	61.0 (58.5–63.2)	37.7 (33.8–41.3)	85.7 (82.3–88.8)	63.3 (59.6–66.6)
Lean NAFLD	0.790 (0.689–0.891)	54.5 (34.6–73.1)	76.1 (71.3–80.5)	35.3 (22.4–47.3)	87.5 (82.0–92.6)	71.9 (64.2–79.1)
Non-lean NAFLD	0.755 (0.705–0.805)	72.7 (65.0–79.6)	56.8 (54.0–59.3)	38.1 (34.0–41.7)	85.1 (80.8–88.8)	61.1 (56.9–64.7)

Abbreviations: AUROC: Area under receiver operating characteristic, BMI: Body mass index, CI: Confidence interval, FIB-4: Fibrosis-4 index, NAFLD: Non-alcoholic fatty liver disease, NFS: Non-alcoholic fatty liver disease fibrosis score, NPV: Negative predictive value, PPV: Positive predictive value.

© 2023 Park H et al. *JAMA Network Open.*

eTable 2. Comparison of the Diagnostic Performance of the FIB-4 and NFS for Diagnosing Individuals With Advanced Hepatic Fibrosis at Current Cut-Off Values Between Nondiabetic and Diabetic Individuals

	Diagnostic performance (95%CI)	Non-diabetic n = 334 (59.0)	Diabetic n = 232 (41.0)	*P-value*
FIB-4	AUROC	0.760 (0.699–0.821)	0.731 (0.661–0.801)	0.54
	Sensitivity	79.1 (68.3–87.4)	86.8 (78.2–92.9)	0.31
	Specificity	57.7 (55.0–59.8)	42.3 (38.1–45.3)	0.003
	PPV	31.9 (27.6–35.3)	42.3 (38.1–45.3)	0.06
	NPV	91.7 (87.3–95.0)	86.8 (78.2–92.9)	0.34
NFS	AUROC	0.758 (0.696–0.819)	0.747 (0.680–0.814)	0.81
	Sensitivity	59.7 (48.4–70.1)	78.9 (69.7–86.5)	0.02
	Specificity	72.7 (69.8–75.3)	41.0 (36.5–44.7)	< 0.001
	PPV	35.4 (28.7–41.6)	39.5 (34.8–43.3)	0.58
	NPV	87.8 (84.4–90.9)	80.0 (71.2–87.2)	0.12

Abbreviations: AUROC: Area under receiver operating characteristic, BMI: Body mass index, CI: Confidence interval, FIB-4: Fibrosis-4 index, NAFLD: Non-alcoholic fatty liver disease, NFS: Non-alcoholic fatty liver disease fibrosis score, NPV: Negative predictive value, PPV: Positive predictive value.

© 2023 Park H et al. *JAMA Network Open.*

eTable 3. Baseline Characteristics of Patients With Biopsy-Proven NAFLD in an Age- and Sex-Matched Cohort According to BMI Quartiles

Characteristics	BMI				*P-value*
	1/4 Quartile (n = 142)	2/4 Quartile (n = 141)	3/4 Quartile (n = 142)	4/4 Quartile (n = 141)	
Age (years)†	53.5 ± 14.9	57.5 ± 12.3	53.2 ± 13.3	44.8 ± 16.9	<.001
Male	59 (41.5)	56 (39.7)	61 (43.0)	57 (40.4)	.95
Female	83 (58.5)	85 (60.3)	81 (57.0)	84 (59.6)	.95
BMI (kg/m^2)†	21.7 ± 1.7	25.3 ± 0.7	28.5 ± 0.9	34.3 ± 4.8	<.001
Glucose (mg/dL)†	117 ± 40	115 ± 33	116 ± 30	117 ± 35	.97
Impaired fasting glucose	85 (59.9)	90 (63.8)	96 (67.6)	96 (68.1)	.43
Diabetes	50 (35.2)	62 (44.0)	59 (41.5)	61 (43.3)	.42
AST (IU/L)†	56 ± 46	70 ± 51	64 ± 37	70 ± 56	.06
ALT (IU/L)†	70 ± 66	91 ± 85	84 ± 56	83 ± 69	.08
AST/ALT†	0.97 ± 0.46	0.90 ± 0.37	0.94 ± 0.60	0.99 ± 0.56	.48
Albumin (g/dL)†	4.26 ± 0.57	4.33 ± 0.34	4.35 ± 0.39	4.21 ± 0.46	.03
Platelets (x10^9/L)†	214 ± 75	215 ± 61	217 ± 69	233 ± 75	.07
FIB-4 index†	2.10 ± 1.78	2.34 ± 1.77	2.13 ± 1.69	1.76 ± 1.44	.03
NAFLD fibrosis score†	-1.62 ± 1.75	-1.20 ± 1.37	-1.01 ± 1.65	-0.84 ± 1.76	0.001
Pathologic diagnosis					N/A
Unknown or borderline	15 (10.6)	11 (7.8)	13 (9.2)	11 (7.8)	
NAFL	47 (33.1)	23 (16.3)	14 (9.9)	24 (17.0)	
NASH	80 (56.3)	107 (75.9)	115 (81.0)	106 (75.2)	
Liver fibrosis by histology					.003
F0	39 (27.5)	19 (13.5)	17 (12.0)	16 (11.3)	
F1	54 (38.0)	47 (33.3)	50 (35.2)	49 (34.8)	
F2	21 (14.8)	39 (27.7)	38 (26.8)	34 (24.1)	
F3	22 (15.5)	30 (21.3)	24 (16.9)	34 (24.1)	
F4	6 (4.2)	6 (4.3)	13 (9.2)	8 (5.7)	
Advanced fibrosis (≥ F3)	28 (25.1)	36 (25.5)	37 (26.1)	42 (29.8)	.27

Data are expressed as number (percent).
†Data are shown as mean ± standard deviation.
Abbreviations: AST, aspartate transaminase; ALT, alanine transaminase; BMI, body mass index; FIB-4 index, fibrosis-4 index; NAFL, non-alcoholic fatty liver; NAFLD, non-alcoholic fatty liver disease; NASH. non-alcoholic steatohepatitis; NFS, NAFLD fibrosis score.

© 2023 Park H et al. *JAMA Network Open.*

eReferences

1. Chalasani N, Younossi Z, Lavine JE, et al. The diagnosis and management of nonalcoholic fatty liver disease: practice guidance from the American Association for the Study of Liver Diseases. *Hepatology*. 2018;67(1):328-357.

2. Liver EAftSoT, Diabetes EAftSo. EASL-EASD-EASO Clinical Practice Guidelines for the management of non-alcoholic fatty liver disease. *Obesity facts*. 2016;9(2):65-90.

3. Kang SH, Lee HW, Yoo J-J, et al. KASL clinical practice guidelines: Management of nonalcoholic fatty liver disease. *Clinical and molecular hepatology*. 2021;27(3):363.

© 2023 Park H et al. *JAMA Network Open.*
