# Supplementary material for: Diagnostic Performance of the Fibrosis-4 Index and Nonalcoholic Fatty Liver Disease Fibrosis Score in Lean Adults With Nonalcoholic Fatty Liver Disease
Source: JAMA Netw Open. 2023 Aug 17;6(8):e2329568. doi: 10.1001/jamanetworkopen.2023.29568 (PMC10436134; doi:10.1001/jamanetworkopen.2023.29568)
Supplement: Supplement 2. — Data Sharing Statement [file jamanetwopen-e2329568-s002.pdf]

Data Sharing Statement

Park. Diagnostic Performance of the Fibrosis-4 Index and Nonalcoholic Fatty Liver Disease Fibrosis Score in Lean Adults With Nonalcoholic Fatty Liver Disease. *JAMA Netw Open*. Published August 17, 2023. doi:10.1001/jamanetworkopen.2023.29568

Data
Data available: No
